# Identification of putative baroreceptors in human aortic arch by histological and omics analyses

**DOI:** 10.1038/s41440-025-02217-9

**Published:** 2025-05-07

**Authors:** Yankey Yundung, Jaroslav Pelisek, Umberto Maccio, Zsuzsanna Varga, Houria Ech-Cherif, Enni Markkanen, Fabian Rössler, Ahmed Ouda, Omer Dzemali, Alexander Zimmermann, Benedikt Reutersberg

**Affiliations:** 1 Department of Vascular Surgery, University Hospital Zurich, Zurich, Switzerland; 2 Department of Pathology and Molecular Pathology, University Hospital Zurich, Zurich, Switzerland; 3 Institute of Veterinary Pharmacology and Toxicology, Vetsuisse Faculty, University of Zurich, Zurich, Switzerland; 4 Department of Visceral Surgery and Transplantation, University Hospital Zurich, Zurich, Switzerland; 5 Department of Cardiac Surgery, University Hospital Zurich, Zurich, Switzerland

**Keywords:** Aortic arch, Aorta, Baroreceptors, Ion channels, PIEZO1, TRPV2, TRPM4, Implemental hypertension

## Abstract

Baroreflex regulates blood pressure and heartbeat through specific mechanosensitive baroreceptors. However, the current knowledge is derived only from animal experiments. No data about human aortic baroreceptors have been reported so far. Therefore, in this study, we performed extended histological, proteomics and transcriptomics analyses of the aortic arch to identify potential human baroreceptors. Three healthy human aortic arches from autopsies, six abdominal aortic aneurysms and four control abdominal aortic tissue samples from our vascular biobank were analysed. For histological analyses, antibodies against various neuronal markers were used. Laser capture microdissection and macrodissection were performed to selectively analyse nerves in the adventitia of the human aorta using proteomics and RNA sequencing. Histological analysis revealed a heterogeneous distribution of nerves in the adventitia along the entire aortic arch, predominantly in the ascending aorta up to the left subclavian artery. Proteome analysis identified three putative human baroreceptors PIEZO1, TRPV2, and TRPM4. Transcriptomics confirmed that these ion channels do not originate from cells within the aortic wall but presumably from the cell body of the vagus nerve. Interestingly, these ion channels were also detected in the healthy abdominal aorta and abdominal aneurysm without any significant differences in their abundance. Our study identified, for the first time, putative baroreceptors in the human aortic arch. Further studies are necessary to validate our current results and elucidate the role of these putative baroreceptors in the human aortic arch.

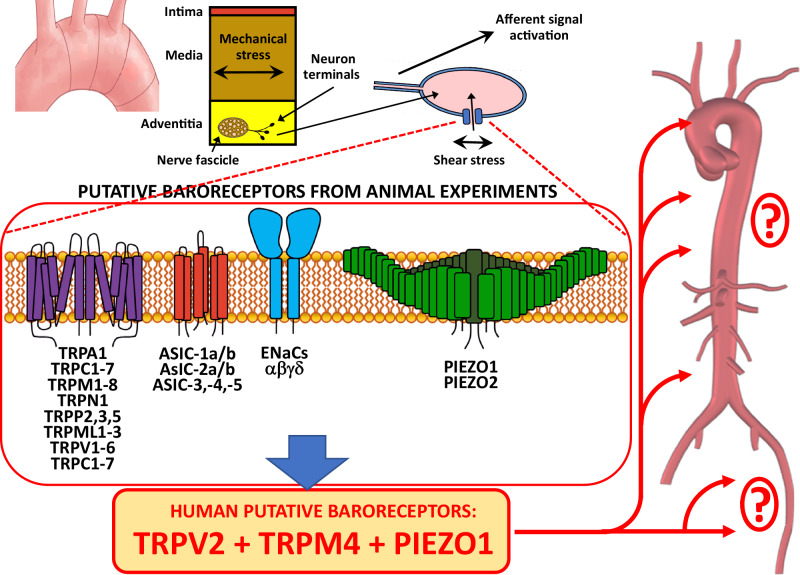

## Introduction

Baroreflex describes the principal neuronal regulatory mechanism of negative feedback control of cardiovascular function by the central nervous system [1, 2]. The heartbeat and blood pressure are regulated in response to the activity of specific arterial baroreceptors located in the carotid sinus and aortic arch [2]. The cell bodies of carotid baroreceptors are located in the petrosal ganglia, and the central process terminates in the nucleus of the solitary tract (NTS). In the case of aortic baroreceptors, the cell bodies are located in the nodose ganglia, and the peripheral process forms part of the vagus nerve, innervating the aortic arch and terminating in the NTS [2, 3]. Increased blood pressure leads to increased activity of the neurons containing mechanosensitive ion channels called baroreceptors, which in turn leads to increased parasympathetic activity, slowing down the heartbeat through the pacemaker cells in the sinoatrial node [3]. Concomitantly, sympathetic activity is inhibited, decreasing the vascular resistance. Thus, baroreflex and baroreceptors are necessary to maintain blood pressure within a physiological range [1–3].

Arterial baroreceptors are located in the plasma membrane of sensory neurons innervating the carotid sinus and aortic arch [4]. Some authors have stated that aortic baroreceptors might be dominant in regulating the heart rate [4–6]. Nothing is known about transmembrane proteins that might serve as putative baroreceptors in the human aortic arch. Thus, it is essential to identify baroreceptors in the human aortic arch to elucidate their contribution to regulating blood pressure in order to endeavour efficient drug therapy. So far, the knowledge of aortic baroreceptors has been derived only from animal models [3, 7, 8]. The animal studies suggest that ion channels such as epithelial sodium (Na^+^) channels (ENaCs), acid-sensing ion channels (ASICs), transient receptor potential (TRP) ion channels, and piezo ion channels are involved in the mechanotransduction performed by arterial baroreceptors [9–19]. All of these ion channels might contribute to arterial baroreflex. However, no data exists on whether these mechanosensitive proteins are expressed in the human aortic arch.

ENaCs and ASICs are members of the amiloride-sensitive degenerin channel (DEG) family and have mainly been suggested to act as mechanosensors [8, 10, 11, 18–20]. Their role and function have been mainly investigated in the carotid sinus [11, 12, 21, 22]. ASIC mRNA and protein have been detected in the aortic baroreceptor terminals in various animal models [8, 11, 13, 16]. TRP ion channels also respond to membrane stretching [8, 13, 15, 16]. TRPV and TRPC have been detected in animal baroreceptor neurons [8, 23, 24]. Recently, novel mechanosensitive ion channels, piezo-type mechanosensitive ion channel component 1 and 2 (PIEZO1 and PIEZO2), have been discovered in mouse neuronal cells, having an important biological function in mechanosensation [9, 18, 19]. The knockout of both PIEZO1 and PIEZO2 in a mouse model was able to completely turn off the baroreceptor reflex [9].

Taken together, numerous ion channels have been assumed to contribute to the arterial baroreflex in animal experiments [8–19]. However, no data exist on whether and which ion channels also play a similar role in the human aortic arch. Therefore, in this study, we performed extended histological, proteomics, and transcriptomics analyses of the healthy aortic arch and abdominal aorta, as well as aortic abdominal aneurysm, in order to identify putative baroreceptors in humans.

## Methods

### Tissue

In this study, various segments from three aortic arches (formalin fixed paraffin embedded (FFPE) tissue: *n* = 6, fresh frozen tissue *n* = 16, including upper and lower part) from the autopsy of patients without cardiovascular comorbidities (Department of Pathology and Molecular Pathology, University Hospital Zurich, Switzerland), six abdominal aortic aneurysms from our vascular biobank (Department of Vascular Surgery, University Hospital Zurich, Switzerland) [25], and four control non-aneurysmal abdominal aortic tissues obtained from the Department of Visceral Surgery and Transplantation (University Hospital Zurich, Switzerland) were used. The local ethics committee approved tissue sample collection and analysis (Cantonal Ethics Committee Zurich, Switzerland; BASEC-Nr. 2020-00378 and 2024-02101). All tissue samples were divided into various segments, some were then directly frozen and stored at −80 °C, whereas others were fixed in 4% formaldehyde for 24 h, decalcified, and embedded in paraffin (FFPE). The entire aortic arch was divided into consecutive circular segments (Supplementary Fig. 1). One of these healthy aortic arches was completely processed for FFPE and used for subsequent histology. For the other two healthy aortic arches, every other segment was processed as FFPE and the rest was fresh-frozen in cryopreservative solution and stored at −80 °C until analysis [25].

### Histology and immunohistochemistry

For histological analysis, FFPE blocks were cut into 5 µm tissue sections, mounted on standard slides (SuperFrost, Thermo Fisher) and conventional stains were applied using haematoxylin-eosin (HE) and Elastica van Gieson (EvG).

For immunohistochemistry (IHC), the tissue sections were mounted on slides coated with poly-L-lysine (SuperFrost Plus, Thermo Fisher) and stained using rabbit or mouse-specific HRP/DAB (ABC) detection IHC kit (abcam) and antibodies against various nerve markers, such as tyrosine hydroxylase (abcam, ab207673, 1:1000), neurofilament (abcam, ab204893, 1:300), protein gene product 9.5 (abcam, ab15503, 1:200), vesicular glutamate transporter 1 and 2 (abcam, ab305253, 1:1000; ab227805, 1:1000; ab211869, 1:50; ab305253, 1:75), brain-derived neurotrophic factor (abcam, ab108319, 1:500), myelin basic protein (abcam, ab133620, 1:300), and calcitonin-gene related peptide (abcam, ab47027, 1:500).

Tyrosine hydroxylase (TH) is present in catecholaminergic neurons, converting tyrosine into L-3,4-dihydroxyphenylalanine (L-DOPA). Protein gene product 9.5 (PGP9.5), also known as ubiquitin C-terminal hydrolase L1, is a deubiquitinating enzyme confined to neurons. Both TH and PGP9.5 are considered general markers of nerves. Neurofilaments (NF) are intermediate filaments within the cytoplasm of neuronal extensions. Vesicular glutamate transporters 1 and 2 (VGLUT 1 and 2, respectively) are glutamate transporters and detect sensory neurons. Brain-derived neurotrophic factor (BDNF) is a member of the neurotrophin family of growth factors found in the peripheral nerves. Myelin basic protein (MBP) shows myelinated nerve fascicles and calcitonin-gene-related peptide (CGRP) is selective for afferent nerves.

Regarding the putative baroreceptors detected by proteomics analysis, the following antibodies were used: anti-piezo-type mechanosensitive ion channel 1 (anti-PIEZO1; Invitrogen, mouse, 1:500), anti-transient receptor potential ion channels V2 and M4 (anti-TRPV2 and anti-TRPM4, Osenses, rabbit, 1:2000 and Origene, mouse, 1:50).

All stained tissue samples were digitalised using ZEISS Axioscan 7 (Zurich, Switzerland). Following digitalisation, the number and area of the nerves within the adventitia were determined using free Software QuPath 0.4.3 (QuPath developers, University of Edinburgh).

### Laser capture microdissection

Laser Capture Microdissection (LCM) was performed as described before [26]. Five FFPE aortic arch tissue blocks from our biobank [25] were cut to a thickness of 10 µm, mounted on PEN membrane glass slides (Thermo Fisher LCM0522), and dried at room temperature overnight. For each sample, four to six tissue sections were used. The slides were stained with cresyl violet, and LCM was performed using the Arcturus Cellect LCM System (Thermo Fisher). From each of the five FFPE tissue samples, nerve fascicles within the adventitia and areas without nerves as controls were excised (Supplementary Fig. 2).

### Macrodissection of FFPE samples

For macrodissection experiments, FFPE samples of abdominal aortic aneurysm (AAA) (*n* = 6), healthy aortic arch (*n* = 6, two segments from each aortic arch), and healthy abdominal aortic tissues (*n* = 4) were used. For each sample, four sections of 20 µm thickness were cut on a microtome (HM340E, Thermo Fisher) and mounted on standard microscope slides. The tunica adventitia was dissected under a light microscope (AE2000, Motic) with a scalpel and transferred to a 1.5 ml Eppendorf tube.

### Macrodissection of fresh samples

Four segments were used, each from two fresh frozen aortic arch samples. In addition, the outer and inner curvatures were treated separately for each segment. The adventitial layer was scraped of the arterial vessel using a scalpel. The proteins from the 16 fresh tissue samples were isolated with RIPA buffer (Thermo Fisher) and total RNA was extracted with TRIZOL reagent (Agilent).

### Proteomics

Proteome analysis was conducted by the Functional Genomics Center Zurich, Proteomics Group (Dr Paolo Nanni). For the samples obtained from LCM, areas of interest on the FFPE slides were identified and transferred onto the LCM cap. After excision, the tissue was inserted into a 1.5 ml centrifuge tube and frozen at −20 °C until further processing. The excised tissue was treated with a commercial proteomics kit (Thermo Fisher). The tissue obtained from macrodissection (FFPE samples) was lysed with 4% SDS and heated at 95 °C for 60 min. High-intensity ultrasound was applied for tissue disruption, and the samples were again heated at 95 °C for 30 min. The following steps were the same way for proteins obtained from macrodissection of the fresh-frozen samples using RIPA buffer, as these did not require tissue lysis because the protein was already isolated. This included the addition of Tris(2-carboxyethyl)phosphine and 2-chloroacetamide to reduce and alkylate the proteins. The samples were incubated for 30 min at 30 °C and diluted with pure ethanol to a final concentration of 60%. Carboxylated magnetic beads were added, and after 30 min at room temperature, the beads were washed thrice with 80% ethanol. Trypsin in 50 mM tetra-ethyl-ammonium bromide (TEAB) was then added for enzymatic digestion overnight at 37 °C. The remaining peptides were extracted from the beads with H_2_O.

For all proteomics experiments (LCM, macrodissection with fresh as well as FFPE samples), the samples were loaded on EvoTip columns (Evosep Biosystem, Denmark), and proteomics was performed on an Evosep One LC coupled to a TIMS TOF Pro mass spectrometer (Functional Genomics Center Zurich (FGCZ), Switzerland). Analysis was performed using DIA-NN software, and only proteins with a number of peptides >1 were included. For the LCM proteomics data, classification and pathway analysis were conducted with the open PANTHER database [27, 28].

### RNA extraction and transcriptomics

The RNA was isolated from the adventitia of the same fresh-frozen tissue used for proteomics, applying TRIZOL reagent (Thermo Fisher) according to the manufacturer’s protocol. The RNA quality and degree of degradation were determined on the TapeStation 4150 (Agilent) using the corresponding RNA ScreenTape Assay in accordance with the manufacturer’s protocols. Library preparation and RNA sequencing were conducted by the Functional Genomics Center Zurich, Transcriptomics Group, using paired-end sequencing on NovaSeq X (Illumina). Raw sequencing data were provided by the SUSHI interface [29]. Further processing steps in SUSHI included the use of several packages such as FastQC for quality control of the raw sequencing data, STAR to align the reads to a reference genome, FeatureCounts to estimate read abundance, and CountQC for quality control after counting reads.

### Data analysis

For differential expression analysis, the linear model approach from the Bioconductor package DESeq2 and EdgeR2 was used [29, 30]. The Benjamini–Hochberg algorithm was used for multiple testing corrections by calculating the False Discovery Rate (FDR, adjusted *P*-value) [31]. All other statistical analyses were performed using R software v4.4.1 or IBM’s SPSS software version 29 (SPSS Inc., Chicago, IL, USA). An independent t-test and a Levene test of equality of variances were used. All statistical analyses were two-sided, with *P* < 0.05 as the significance level.

## Results

### Histological and immunohistochemical analysis of peripheral nerves

**Fig. 1:**
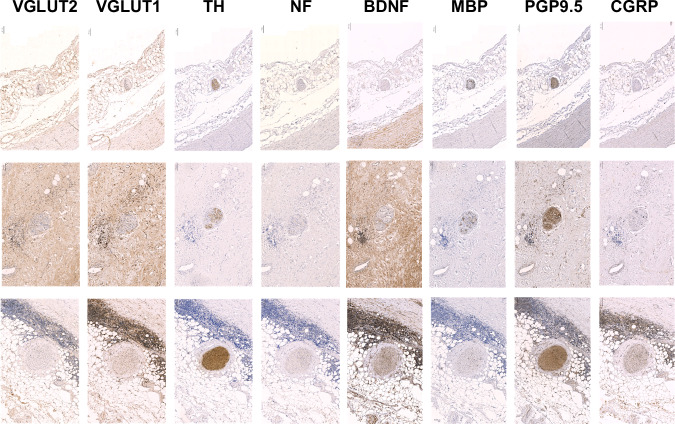
Optimised immunohistochemical staining of nerves in the human aortic arch (*n* = 3). Different antibodies were used against various types of peripheral nerves. VGLUT1 and 2 vesicular glutamate transporter 1 and 2 (dilution 1:1000 and 1:75), TH tyrosine hydroxylase (dilution 1:1000), NF neurofilament (dilution 1:300), BDNF brain-derived neurotrophic factor (dilution 1:500), MBP myelin basic protein (dilution 1:300), PGP9.5 protein gene product 9.5 (dilution 1:200), CGRP calcitonin gene-related peptide (dilution 1:500)

**Fig. 2:**
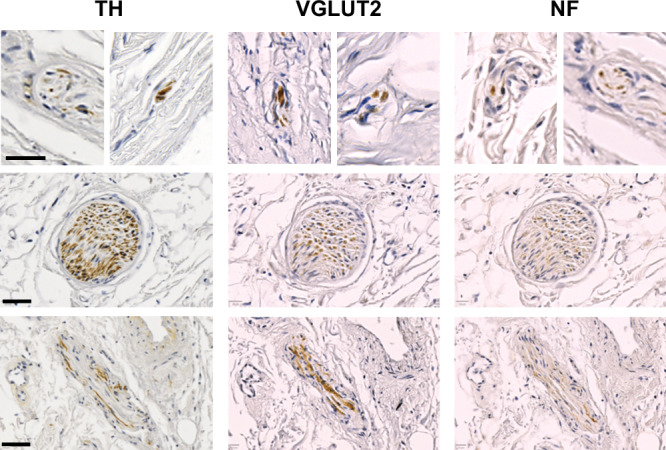
Selected immunohistochemical staining of nerves of various sizes in the human aortic arch. Antibodies against tyrosine hydroxylase (TH, 1:1000)) neurofilament (NF, 1:300)) and vesicular glutamate transporter 2 (VGLUT2, 1:75) on consecutive slides were used. Scale bars 50 µm

First, several antibodies were applied and optimised in order to detect the different types of peripheral nerves (Fig. 1). Under optimised conditions (antigen retrieval solution and pH, antibody dilution and incubation time), anti-TH and anti-PGP9.5 were able to detect all nerves within the arterial wall. However, TH appeared to have less background than PGP9.5. The antibody against BDNF could not be optimised appropriately, as it has a heavy background independent of the staining conditions. The staining against VGLUT1 also had a strong background, with almost all cells positive. The antibody staining against CGRP was weak and unspecific and could not be clearly assigned to the nerves. The staining of MBP worked well, however, we found only sporadic spots within the aortic wall. Following an extended immunohistochemical optimisation of various peripheral nerves and nerve endings in the human aorta and aortic arch, TH, NF and VGLUT2 were chosen, being able to unambiguously stain nerves in the human aortic arch (Fig. 2). We detected not only large nerve fascicles but also individual nerve fibres. Some of the TH-positive nerve fascicles had various diameters of up to 760 µm. Furthermore, several of these nerve fascicles ran alongside the blood vessel and thus had a round shape. Others, running circumferential, had an elongated or linear form (Fig. 2).

**Fig. 3:**
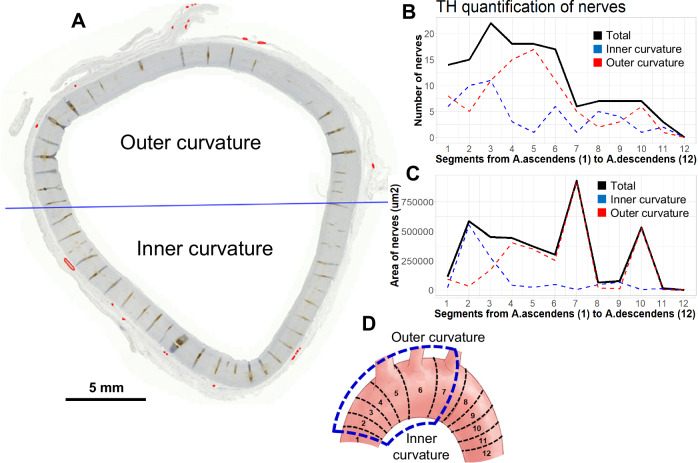
Quantification of nerve distribution along the aortic arch using tyrosine hydroxylase (TH) staining. A Example of the image quantification. The blue line indicates the separation between the outer and inner curvature. The nerves (TH-positive staining) are indicated by red lines. **B** Quantification plot showing the number of nerves in the individual segments (see Supplementary Fig. 1). **C** Quantification plot focusing on the TH-stained area. **D** A schematic image of the aortic arch with the individual segments analysed in the study. The blue dashed line displays the part of the aortic arch with the most nerves and nerve endings

In order to gain a more detailed insight into the frequency of nerve appearance within the human aortic arch, quantitative analysis was conducted by counting the number of TH-positive nerves (Fig. 3A, B). Furthermore, the TH-stained areas were determined (Fig. 3C). Some segments (5–7, see Fig. 3D and Supplementary Fig. 1) also contained parts of the brachiocephalic, left common carotid and left subclavian arteries. These selective parts were excluded from the analysis, focusing only on the aortic arch (Supplementary Fig. 3). Some segments (particularly segments 8–12) contained very few nerves. However, the detected nerve fascicles were markedly larger than those in the other segments. This was particularly visible when calculating the TH-positive areas (Fig. 3C). Segments 1–6, which are part of the ascending aorta until the left common carotid artery, seemed to have markedly more nerves than the remaining segments 7–12, starting with the left subclavian artery towards the descending aorta. Interestingly, no differences in nerve distribution were observed between the lower and upper aortic arch.

### Laser capture microdissection

**Fig. 4:**
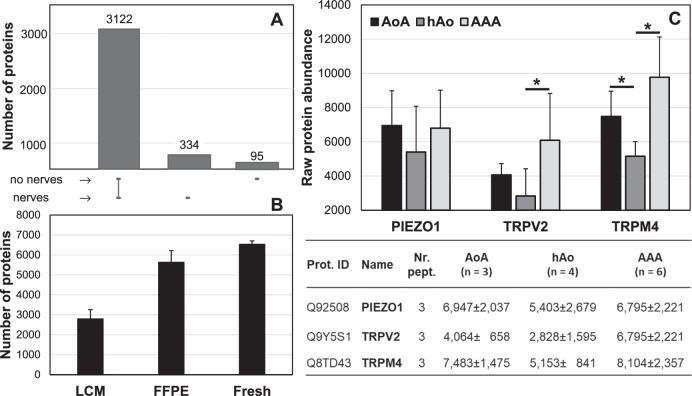
Proteomics. **A** Proteome analysis - Diagram showing the total number of proteins present in the LCM samples and the number of proteins found only in the group with nerves and without nerves. **B** Number of proteins detected in the various experimental approaches: LCM (laser capture microdissection), FFPE (macrodissection from formalin-fixed in paraffin-embedded samples), and Fresh (fresh frozen tissue samples). **C** Proteome analysis - raw protein abundance in the adventitia of the FFPE aortic tissue samples. PIEZO1 piezo-type mechanosensitive ion channel 1, TRPV2 and TRPM4 transient receptor potential ion channels V2 and M4, AoA aortic arch, hAo healthy abdominal aorta, AAA abdominal aortic aneurysm. The table shows the mean ± standard deviation of the individual detected putative mechanosensitive ion channels

As shown in Supplementary Fig. 2, nerve fascicles and the neighbouring area without nerves were removed using Laser Capture Microdissection and analysed by proteomics. In total, more than 3000 proteins were identified using LCM (Fig. 4A). Interestingly, 334 proteins were found to be significantly more abundant in the nerves than in the control adventitial tissue (FDR < 0.05, log2FC threshold of 0.5, number of peptides >1). In this group, many neuron-specific proteins, such as neurofilament polypeptides, neural cell adhesion molecules, peripherin, and Schwann cell-specific proteins like myelin-related proteins, were identified. These 334 significantly abundant proteins in the nerves were further analysed in the PANTHER database according to their molecular function, biological process, cellular component, protein class and pathway. In the cellular compartment analysis, 9 proteins were found to belong to the axon, 8 to synapses, 9 to neuron projections and 6 to the neuronal cell body (Supplementary Table 1). In addition, in the biological process analysis, processes belonging to the nervous system were present, such as axonogenesis, chemical synaptic transmission, myelination, neuron projection development, synaptic signalling, and synapse organisations (Supplementary Table 2). For the pathway analysis, serotonin receptor-mediated signalling, serotonin degradation, adrenaline and noradrenaline biosynthesis, alpha and beta-adrenergic receptor signalling, axon guidance, dopamine receptor-mediated signalling pathway, GABA receptor signalling, ionotropic and metabotropic glutamate receptors, muscarinic and nicotinic acetylcholine receptor and synaptic vesicle trafficking pathways were implicated (Supplementary Table 3).

Surprisingly, using laser capture microdissection, we did not identify any baroreceptor candidates in the nerve areas or surrounding regions.

### Proteomics of adventitia from macrodissected FFPE aortic tissue samples

As described above, LCM did not lead to the detection of any potential mechanosensitive receptors in the nerves within adventitia. However, because several publications on animal models have described baroreceptors in the aortic arch, we analysed in the next step the entire adventitia [4, 7, 8, 11, 12, 16].

In order to compare the protein expression also in other sections of human aortas, we performed a macrodissection of FFPE tissue samples from the healthy aortic arch, healthy abdominal aorta and abdominal aortic aneurysm. Using whole adventitia, 5.631 ± 584 proteins from FFPE specimens could be identified (Fig. 4B). Interestingly, among these proteins, three putative baroreceptors with a number of peptides >1 were detected: PIEZO1, TRPM4 and TRPV2 (Fig. 4C). Surprisingly, these ion channels and possible baroreceptor candidates were found not only in the aortic arch but also in the adventitia of the healthy abdominal aorta as well as in the abdominal aortic aneurysm. Considering the protein abundance (Fig. 4C and the corresponding table), PIEZO1 had similar protein abundance in all three study groups (Fig. 4C). In contrast, a significantly increased amount of TRPV2 was observed in AAA compared to the aortic arch or healthy aorta. For TRPM4, the lowest protein abundance was observed in the abdominal aorta in the absence of cardiovascular comorbidities (Fig. 4C).

### Proteomics of adventitia from macrodissected fresh aortic arch samples

In order to further improve the sensitivity of our analysis, we focused on the adventitia of the fresh-frozen samples. Using this approach, we divided the aortic arch into upper and lower segments and analysed these segments separately. Using fresh tissue, over 6000 proteins were identified by proteome analysis (Fig. 4B). Considering the number of peptides >1, the same putative baroreceptors, PIEZO1, TRPV2 and TRPM4, were also found in the adventitia of fresh frozen aortic tissue samples (Table 1). Interestingly, no significant differences were observed between the protein abundances of the upper and lower halves of the same segment (FDR < 0.05, log2FC threshold of 0.5).

**Table 1:** Raw protein abundance in adventitia of the fresh frozen aortic arch tissue samples of the detected putative baroreceptors

		Nr. of peptides	AoA area	AoA 1/analysed segments^a^				AoA 3/analysed segments^a^					
Protein ID	Name												
				1/1	1/3	1/5	1/7	3/1	3/3	3/5	3/9	Mean ± SD	*P*-value
			Upper	2717	5986	5447	2985	2930	2438	2707	2603	3476 ± 1400	
Q92508	PIEZO1	3											0.202
			Lower	2740	3219	3678	3980	1154	2372	1815	2413	2671 ±943	
			Upper	1566	2720	1917	3925	609	1211	789	1561	1787 ± 1087	
Q9Y5S1	TRPV2	3											0.673
			Lower	1290	3098	2351	1182	1213	659	1274	1604	1584 ±776	
			Upper	1700	2797	2253	3657	4817	5562	4961	3607	3669 ± 1374	
Q8TD43	TRPM4	3											0.438
			Lower	2830	2128	1735	1716	4425	4108	4483	3815	3155 ± 1193	

*PIEZO1* piezo-type mechanosensitive ion channel 1, *TRPV2 and TRPM4* transient receptor potential ion channels V2 and M4, *AoA* aortic arch, *SD* standard deviation

^a^Individual segment of two human healthy aortic arches (see also Fig. 1 and Fig. 3D)

### Transcriptomics of adventitia from the macrodissected fresh aortic arch samples

Finally, transcriptome analysis of the same fresh frozen aortic arch tissue samples used in proteomics was performed. Except for ASIC4 and the alpha-subunit of ENaC, all other expressed mRNAs of the potential mechanosensitive proteins were members of the transient receptor potential ion channel family (Table 2). Particularly high expression was observed for TRPP2. On the contrary, low expression was observed for TRPC3, TRPV5, and the mucolipin TRP channel MCOLN3. Similar to the comparison of the protein abundances, no significant differential expression of the detected ion channel genes between the upper and lower part of the aortic arch (FDR < 0.05, log2FC threshold of 0.5) was observed.

**Table 2:** Transcriptome analysis – normalised RNA expression (RPKM = reads per kilobase of transcript per million) of various ion channels that might serve as putative baroreceptors in the adventitia of the fresh frozen aortic arch tissue samples

Gene ID	Gene name	AoA area	AoA 1/analysed segments				AoA 3/analysed segments					
			1/1	1/3	1/5	1/7	3/1	3/3	3/5	3/9	Mean ± SD^a^	*P*-value
ENSG00000072182	ASIC4	Upper	20.7	2.2	1.7	2.3	4.0	12.3	2.8	8.2	6.8 ± 6.7	0.674
		Lower	4.5	5.4	4.0	–	5.7	10.6	1.8	7.2	5.6 ± 2.8	
ENSG00000162572	SCNN1D	Upper	20.6	9.9	2.5	7.2	10.3	24.2	8.2	15.4	12.3 ± 7.3	0.539
		Lower	24.1	23.0	8.7	14.6	13.4	8.4	6.5	17.1	14.5 ± 6.6	
ENSG00000104321	TRPA1	Upper	17.8	2.3	1.9	4.2	8.1	8.6	8.7	6.7	7.3 ± 5.1	0.906
		Lower	9.5	7.5	0.9	5.7	6.8	13.2	8.9	7.9	7.6 ± 3.5	
ENSG00000144935	TRPC1	Upper	4.9	3.6	5.1	5.5	8	6.7	13.5	5	6.5 ± 3.1	0.084
		Lower	3.7	1.8	2.7	1.7	6.6	6.8	5.9	3.4	4.1 ± 2.1	
ENSG00000118762	TRPP2	Upper	40.0	51.2	68.8	64.6	268	244	188	81.1	126 ± 93	0.830
		Lower	52.2	57.2	57.9	33.5	262	209	182	73	115 ± 88	
ENSG00000138741	TRPC3	Upper	8.5	2.6	1.2	–	2.3	1.4	4.6	4.1	3.5 ± 2.5	0.969
		Lower	3.0	2.2	1.8	–	5.9	3.9	4.2	3.4	3.4 ± 1.4	
ENSG00000069018	TRPC7	Upper	20.6	0.5	2.3	1.5	0.6	3.9	6	5.3	5.1 ± 6.6	0.929
		Lower	2.2	–	1.3	–	7.8	8	5.3	4.5	4.9 ± 2.8	
ENSG00000083067	TRPM3	Upper	33.6	5.2	2.4	2.8	11.2	5.4	17	9.3	10.9 ± 9.7	0.969
		Lower	8.3	5.1	7.0	7.5	12.7	18	11	16	10.7 ± 4.6	
ENSG00000119121	TRPM6	Upper	15.0	1.9	1.8	2.0	5.8	4.5	8.8	4.8	5.6 ± 4.5	0.896
		Lower	4.0	2.8	1.8	5.4	5.7	15.1	4.8	7.3	5.9 ± 4.1	
ENSG00000144481	TRPM8	Upper	38.6	5.5	2.4	2.1	8.9	17.2	7.8	12.5	11.9 ± 9.4	0.897
		Lower	6.4	13.7	2.8	11.6	10.2	17.5	12.8	15.2	11.3 ± 4.8	
ENSG00000127412	TRPV5	Upper	11.5	–	–	–	0.6	–	2.1	0.7	3.7 ± 5.2	0.289
		Lower	1.1	–	0.7	2.1	1.6	2.4	0.9	2.1	1.6 ± 0.7	
ENSG00000165125	TRPV6	Upper	21.4	2.1	2.0	0.7	9.5	9.9	12	8.6	8.3 ± 6.8	0.918
		Lower	6.6	4.2	5.2	2.0	7	17.2	10.6	16	8.6 ± 5.5	
ENSG00000055732	MCOLN3	Upper	8.7	1.5	2.3	0.4	3.2	–	1.9	5.1	3.3 ± 2.8	0.785
		Lower	1.0	4.0	0.6	–	4.5	3.3	2.5	4.8	3.0 ± 1.7	

*ASIC4* Acid-sensing ion channel 4, *SCNN1D* alpha subunit of the epithelial sodium channel (ENaC), *TRP* transient receptor potential cation channels, *MCOLN3* Mucolipin TRP cation channel 3

^a^Mean ± standard deviation

Interestingly, no expression was found for the three putative baroreceptors PIEZO1, TRPV2 and TRPM4, identified in the proteome analysis.

### Immunohistochemical analysis of the putative baroreceptors PIEZO1, TRPV2 and TRPM4

In order to localise and ascertain the distribution of the above-identified putative baroreceptors in the human aortic arch, an extensive immunohistochemical analysis of PIEZO1, TRPV2 and TRPM4 was performed. However, even if every staining was positive in the control tissue (mouse brain), we were not able to reliably identify any potential baroreceptors in the aortic arch by immunohistochemistry. The reasons for failing to detect these baroreceptors are discussed in the section Discussion.

## Discussion

In the current study, we identified putative baroreceptors that have not yet been described in the human aortic arch. Our results demonstrated a heterogeneous distribution of nerves of different sizes along the aortic arch located in the adventitia, particularly in the ascending aorta and up to the left subclavian artery. There was no accumulation of nerves in the inner curvature of the aortic arch. Using extended proteome analysis, we identified three putative human baroreceptors, PIEZO1, TRPV2 and TRPM4.

Extended IHC analysis of nerves within the human aortic arch wall revealed neuronal fascicles of various sizes exclusively in the adventitia. In particular, an accumulation of nerves was observed in the left subclavian artery, corresponding with the findings in animal models of rabbits and mice [32, 33]. Furthermore, the aortic depressor nerve containing the aortic baroreceptors is located between the left subclavian and the left common carotid artery [8, 18, 33]. Regarding different types of nerves, we were able to detect the sensory nerves using the VGLUT2 antibody. The circumstance that the aortic depressor nerve is appointed between the subclavian and left common carotid arteries also explains the fact that most nerves along the aortic arch were located in the ascending artery up to segment 7 (see also Fig. 3D) [33]. Thus, we assume that the putative human baroreceptors in this area, especidally between left subclavian and left common carotid arteries. However, they are not located exclusively in the inner aortic curvature but alongside the entire circumference of the aortic arch. This assumption is in line with the results of Min et al. [33], who demonstrated the distribution of sensory neurons around the aortic arch by using specific VGLUT2 (marker of sensory neurons) and PIEZO2 transgenic mouse model, showing the branching of the left aortic depressor nerve at the sides of the aortic arch between the left subclavian and common carotid arteries.

Using proteomics of the microdissected nerve fascicles, we did not detect any potential baroreceptors in the aortic arch. We assume that the nerve fascicles, containing a composite of nerve fibres, do not contain these mechanosensitive proteins. They are probably located only at the end of the unmyelinated nerve fibres [3]. In contrast, after analysing the entire adventitia, three putative baroreceptors, PIEZO1, TRPV2, and TRPM4 were detected. These results confirm that the mechanosensitive receptors are not located in the nerve fascicles but in the single nerve fibres, most likely at their terminals. This might be the reason why the immunohistochemical analysis did not identify any of these receptors. Therefore, they are difficult to identify if the staining is not clear and when the antibodies have not yet been established in humans. Only if we were fortunate and cut exactly through these neuronal terminals would we be able to detect these putative baroreceptors also by immunohistochemistry. It is also to mention that the FFPE section are only 5 µm thick, and the nerve terminals have a diameter of about 10 µm. The separate analysis of the inner and outer curvatures of the aortic arch did not demonstrate any significant differences in the abundance of these ion channels. Again, these results confirm the equal distribution of the putative baroreceptors around the aortic arch without any specific hotspots.

We were also interested in the possible presence of these baroreceptors in the abdominal aorta in order to evaluate their specificity for the aortic arch. Therefore, we included healthy abdominal aorta and abdominal aortic aneurysm in the analysis because we have already found an increased number of neuronal fascicles in the diseased aorta [34]. Surprisingly, we detected the putative baroreceptors PIEZO1, TRPV2 and TRPM4 in the abdominal aorta. In addition, no significant differences were observed between healthy and diseased abdominal aorta. Interestingly, only three mechanosensitive receptors have been identified. The consequence of this finding is that PIEZO1, TRPV2 and TRPM4 seem to have a general pattern of appearance along the human aorta. Proteome analysis was performed because nerve endings in the adventitia of the aortic arch do not contain any nuclei as they are located in the brainstem. In order to verify whether these potential baroreceptors are expressed by other cells with their nuclei present in the adventitia of the aorta, we performed an additional transcriptome analysis of the same samples. Interestingly, different baroreceptor candidates, previously described in the animal models, have been identified [9–12, 14, 16, 18]. In particular, various TRP ion channels were detected. It has already been reported that TRP channels are expressed in SMCs and ECs and participate in regulating vascular tone [35, 36]. Aberrant expression of TRP channels was associated with vascular dysfunction and metabolic syndromes. Regarding TRPV2, the expression of this ion channel has already been found in vascular cells, not yet, however, in human vasculature, only in various animal models [37]. The expression of TRPM4 has been detected in e.g. SMCs, however, again, only in animals [38]. The expression of PIEZO channels has been so far associated with cardiovascular development [39]. In contrast, our results demonstrated no expression of PIEZO1, TRPV2 or TRPM4 at the mRNA level in humans. It is not surprising because the cell nuclei of the sensory neurons containing the putative mechanosensitive receptors of the baroreflex are located in the cell bodies within the brain stem. It has been stated that most of the RNA detected in neuron fibres belongs to ribosomes, mitochondria and cytoskeleton [40]. In general, RNAs can be transported along the nerve fibres by RNA-binding proteins (RBPs) [41]. However, the transport efficiency depends on the mRNA lengths and distance from the cell nuclei. All three putative baroreceptors detected in the study have a large mRNA ranging from 2.4 to 7.6 kbp. Thus, we assume that our baroreceptor candidates are either translated in the cell bodies and transported as proteins along the neuronal fibres [42] or the turnover rate for these receptors is so low that no RNA could be detected, even if those would be transcribed in the nerve endings.

Consequently, these three putative baroreceptors are not expressed in any cells within the adventitia of the aortic arch and are presumably associated with nerves whose cellular bodies are in the brainstem. The appearance of these three baroreceptors in the abdominal aorta may lead to the conclusion that these mechanosensitive ion channels are not specific for a particular function, such as, for instance, baroreflex. They are most likely to be expressed in the human aorta. However, little is known about, where the nerves containing the mechanosensitive ion channel lead. In the aortic arch but not in the abdominal aorta, the ion channels transmit probably the signal to the baroreflex centre in the brainstem. The aortic arch is innervated by the aortic depressor nerve and the abdominal aorta is innervated primarily by the abdominal aortic plexus [1, 2, 4].

Regarding mechanosensitive baroreceptors, more than 30 different ion channels have been described in the aortic arch of various animal models [8–10, 13, 14, 17–19, 21], such as ENaCs [10, 12, 43], ASIC [8, 11, 13, 16], TRPs [8, 13, 15, 16], and PIEZOs [9, 18, 19]. Interestingly, in the human aortic arch, we detected only the family of TRP ion channels, namely TRPV2, TRPM4, and PIEZO1 of the recently discovered PIEZO mechanosensitive family of ion channels.

TRPs belong to a large family of cellular sensors responding to various stimuli such as e. g. taste, temperature, osmotic stress, membrane stretching, and others [8, 15, 23, 44, 45]. TRPMs and TRPVs have been identified in various animal models as baroreceptors [8, 15, 46]. The mammalian TRPM family consists of 8 genes, TRPM1-8 (melastatin 1–8). Thereby, TRPM4, detected in our study, was shown to be permeable to monovalent cations and activated by intracellular Ca^2+^ ions, leading to a pronounced voltage modulation [47]. Furthermore, TRPM4 has been described as being able to transduce mechanical stimuli into a change in vascular tone [48]. Other researchers have reported that TRPM4 is activated by membrane stretch [49]. However, the role of TRPM4 in the baroreflex has not yet been described. The TRPV family (vanilloids 1–6) consists of six members. TRPV1-4 are sensitive to various physical stimuli such as stretching, shear stress or osmolarity [45]. They play important role in the regulation of normal and pathological cellular functions in the vasculature. Thereby, TRPV2 has been detected in a wide range of tissues and organs, including neurons, pancreatic β-cells, smooth muscle, and endothelial cells [24]. However, the relevance of TRPV2 and its function in vascular cells, particularly in the context of baroreflex, remain yet to be elucidated.

PIEZO1 was another putative baroreceptor detected in the present study. This ion channel is a promising candidate because PIEZOs have already been described to act as mechanosensitive baroreceptors gated by force, stretch sensation or shear stress [9, 19, 50, 51]. There are two known PIEZO ion channels, PIEZO1 and 2. They have already been described to play various roles in the pathogenesis of hypertension, affecting vascular cells, baroreflex, and the juxtaglomerular apparatus [8, 9, 51]. Furthermore, PIEZO1 has been detected in the cellular membrane, thus being a promising baroreceptor candidate [9]. The knockout of both PIEZO1 and PIEZO2 in a mouse model completely eliminated the baroreceptor reflex. Interestingly, we did not detect PIEZO2 in the human aorta. It seems that in humans, only PIEZO1 is acting as a putative mechanosensitive baroreceptor. In a rat model of hypertension, Cui et al. [52] emphasised the importance of PIEZO1 in decreasing blood pressure by injecting this protein directly into the bloodstream in a rat model of hypertension. Further studies are necessary to elucidate the role of PIEZO1 as a potential baroreceptor in the human aortic arch.

Study Limitations: Obtaining healthy human aortic arch tissue samples without cardiovascular comorbidity is challenging, limiting the sample size in our study to only three individuals, which is relatively small. Therefore, in order to enhance statistical power, we utilised different segments from the available specimens. Consequently, we analysed various segments, including FFPE and fresh-frozen samples (*n* = 6 and 16). The three putative baroreceptors - PIEZO1, TRPV2, and TRPM4 - were detected in all analysed samples and segments. Nevertheless, further research with more specimens and patients is needed to explore the role of these ion channels in baroreflex function and to fully elucidate their functional significance. In the next study, we intend to analyse the expression of these baroreceptors along the entire arterial tree in order to decipher their distribution in all human arteries.

## Conclusions

In this study, we identified for the first time, putative baroreceptors in the human aortic arch. We found an equal distribution of nerves exclusively in the adventitia along the entire aortic arch, predominantly in the ascending aorta up to the left subclavian artery. Through proteome analysis, we identified three potential human baroreceptors PIEZO1, TRPV2, and TRPM4. Using transcriptomics as a process of elimination, no mRNA expression of these three ion channels was detected. Consequently, we hypothesise that these proteins originate from baroreceptor neurons with their cell bodies in the nodose ganglion, which is responsible for the baroreflex. Interestingly, we also found these ion channels in both healthy and diseased abdominal aortas. We assume that PIEZO1, TRPV2, and TRPM4 are generally expressed along the human aorta. Their function, however, is determined by the cell type they are expressed and in the case they are expressed in neurons, their function depends on which path the neuronal fibres take. Further studies are necessary to confirm our current results and determine the extent to which these three ion channels act as baroreceptors in the human aortic arch.

## Supplementary information

Supplementary Table 1

Supplementary Table 2

Supplementary Table 3

Supplementary Figure1

Supplementary Figure2

Supplementary Figure3

Supplementary Figure Legend
