# Supplementary material for: Identification of putative baroreceptors in human aortic arch by histological and omics analyses
Source: Hypertens Res. 2025 May 7;48(7):2083–94. doi: 10.1038/s41440-025-02217-9 (PMC12229889; doi:10.1038/s41440-025-02217-9)
Supplement: Supplementary file 1 — Supplementary Table 1 [file 41440_2025_2217_MOESM1_ESM.docx]

**Supplementary Table 1** PANTHER database analysis of the neuron-related proteins: Cellular Components.

| **Cellular Component** | **Protein Designation** |
| --- | --- |
| Axon | CONTACTIN-1, GLYCOGEN SYNTHASE KINASE-3 BETA, PERIPHERIN, VIMENTIN, TYROSINE 3-MONOOXYGENASE, KINESIN-LIKE PROTEIN KIF1A, NEURAL CELL ADHESION MOLECULE L1, NEUROFILAMENT MEDIUM POLYPEPTIDE, NEUROFILAMENT LIGHT POLYPEPTIDE |
| Axon terminus | GAMMA-SYNUCLEIN, ALPHA-SYNUCLEIN |
| Dendrite | KINESIN-LIKE PROTEIN KIF1A, MICROTUBULE-ASSOCIATED PROTEIN 1B, MICROTUBULE-ASSOCIATED PROTEIN 1A |
| Glutamatergic synapse | GLYCOGEN SYNTHASE KINASE-3 BETA |
| Neuron projection | STATHMIN, MICROTUBULE-ASSOCIATED PROTEIN 4, MICROTUBULE-ASSOCIATED PROTEIN TAU, NEURAL CELL ADHESION MOLECULE 1, BASEMENT MEMBRANE-SPECIFIC HEPARAN SULFATE PROTEOGLYCAN CORE PROTEIN, NEURAL CELL ADHESION MOLECULE L1-LIKE PROTEIN, ANKYRIN-3, ANKYRIN-2, CELL ADHESION MOLECULE 1 |
| Neuronal cell body | GAMMA-SYNUCLEIN, ALPHA-SYNUCLEIN, MICROTUBULE-ASSOCIATED PROTEIN 1B, MICROTUBULE- ASSOCIATED PROTEIN 1A, NEURAL CELL ADHESION MOLECULE L1, HEAT SHOCK PROTEIN HSP 90-ALPHA |
| Postsynapse | ALPHA-INTERNEXIN, NEUROFILAMENT MEDIUM POLYPEPTIDE, NEUROFILAMENT LIGHT POLYPEPTIDE |
| Postsynaptic membrane | BTB_POZ DOMAIN-CONTAINING PROTEIN KCTD12 |
| Presynaptic membane | BTB_POZ DOMAIN-CONTAINING PROTEIN KCTD12 |
| SNARE complex | SYNAPTOSOMAL-ASSOCIATED PROTEIN 25 |
| Synapse | GLYPICAN-1, DYNAMIN-1, MICROTUBULE-ASSOCIATED PROTEIN 1B, MICROTUBULE-ASSOCIATED PROTEIN 1A, INTEGRIN BETA-1, BETA-1-SYNTROPHIN, CELL ADHESION MOLECULE 1, DYSTROBREVIN ALPHA |
