# Supplementary material for: Identification of putative baroreceptors in human aortic arch by histological and omics analyses
Source: Hypertens Res. 2025 May 7;48(7):2083–94. doi: 10.1038/s41440-025-02217-9 (PMC12229889; doi:10.1038/s41440-025-02217-9)
Supplement: Supplementary file 2 — Supplementary Table 2 [file 41440_2025_2217_MOESM2_ESM.docx]

**Supplementary Table 2** PANTHER database analysis of the neuron-related proteins: Biological Processes.

| **Biological Process** | **Protein designation** |
| --- | --- |
| Axo-dendritic transport | KINESIN-LIKE PROTEIN KIF1A |
| Axon development | MYELIN PROTEOLIPID PROTEIN |
| Axon guidance | CONTACTIN-1 |
| Axonogenesis | MICROTUBULE-ASSOCIATED PROTEIN 1A, MICROTUBULE-ASSOCIATED PROTEIN 1B |
| Brain development | CONTACTIN-1 |
| Catecholamine metabolic process | DOPAMINE BETA-HYDROXYLASE, TYROSINE 3-MONOOXYGENASE |
| Central nervous system development | MYRISTOYLATED ALANINE-RICH C-KINASE SUBSTRATE |
| Chemical synaptic transmission | ALPHA-SYNUCLEIN, GAMMA-SYNUCLEIN |
| Dendrite development | MICROTUBULE-ASSOCIATED PROTEIN 1A, MICROTUBULE-ASSOCIATED PROTEIN 1B |
| Dopamine receptor signaling pathway | GUANINE NUCLEOTIDE-BINDING PROTEIN G(O) SUBUNIT ALPHA |
| Glial cell differentiation | PERIAXIN |
| Glutamate metabolic process | GLUTAMINASE KIDNEY ISOFORM (MITOCHONDRIAL) |
| Myelination | PERIAXIN, MYELIN PROTEOLIPID PROTEIN, MYELIN PROTEIN P0 |
| Neuron development | DYSTROPHIN |
| Neuron projection development | STATHMIN, MICROTUBULE-ASSOCIATED PROTEIN TAU, MICROTUBULE-ASSOC. PROTEIN 4 |
| Neurotransmitter secretion | SYNTAXIN-BINDING PROTEIN 1, SYNTAXIN-BINDING PROTEIN 3 |
| Oligodendrocyte differentiation | MYELIN PROTEOLIPID PROTEIN |
| Peripheral nervous system development | PERIAXIN |
| Regulation of neuron development | GLYCOGEN SYNTHASE KINASE-3 BETA |
| Synapse organization | ALPHA-SYNUCLEIN, GAMMA-SYNUCLEIN, NEURAL CELL ADHESION MOLECULE L1 |
| Synaptic membrane adhesion | RECEPTOR-TYPE TYROSINE-PROTEIN PHOSPHATASE F |
| Synaptic signaling | DYSTROPHIN, DYSTROBREVIN ALPHA |
| Synaptic vesicle endocytosis | DYNAMIN-1, ALPHA-SYNUCLEIN, GAMMA-SYNUCLEIN |
| Synaptic vesicle exocytosis | SYNAPTOSOMAL-ASSOCIATED PROTEIN 25 |
| Synaptic vesicle transport | DYNAMIN-1 |
