# Supplementary material for: Identification of putative baroreceptors in human aortic arch by histological and omics analyses
Source: Hypertens Res. 2025 May 7;48(7):2083–94. doi: 10.1038/s41440-025-02217-9 (PMC12229889; doi:10.1038/s41440-025-02217-9)
Supplement: Supplementary file 3 — Supplementary Table 3 [file 41440_2025_2217_MOESM3_ESM.docx]

**Supplementary Table 3** PANTHER database: Pathway analysis.

| **Pathway ID** | **Pathway Designation** |
| --- | --- |
| P04373 | 5HT1 type receptor mediated signaling pathway |
| P04374 | 5HT2 type receptor mediated signaling pathway |
| P04375 | 5HT3 type receptor mediated signaling pathway |
| P04376 | 5HT4 type receptor mediated signaling pathway |
| P04372 | 5-Hydroxytryptamine degredation |
| P00001 | Adrenaline and noradrenaline biosynthesis |
| P00002 | Alpha adrenergic receptor signaling pathway |
| P00009 | Axon guidance mediated by netrin |
| P00007 | Axon guidance mediated by semaphorins |
| P04377 | Beta1 adrenergic receptor signaling pathway |
| P04378 | Beta2 adrenergic receptor signaling pathway |
| P04379 | Beta3 adrenergic receptor signaling pathway |
| P04380 | Cortocotropin releasing factor receptor signaling pathway |
| P05912 | Dopamine receptor mediated signaling pathway |
| P05730 | Endogenous_cannabinoid_signaling |
| P05913 | Enkephalin release |
| P05731 | GABA-B_receptor_II_signaling |
| P00037 | Ionotropic glutamate receptor pathway |
| P00041 | Metabotropic glutamate receptor group I pathway |
| P00040 | Metabotropic glutamate receptor group II pathway |
| P00039 | Metabotropic glutamate receptor group III pathway |
| P00042 | Muscarinic acetylcholine receptor 1 and 3 signaling pathway |
| P00043 | Muscarinic acetylcholine receptor 2 and 4 signaling pathway |
| P06587 | Nicotine pharmacodynamics pathway |
| P00044 | Nicotinic acetylcholine receptor signaling pathway |
| P05916 | Opioid prodynorphin pathway |
| P05915 | Opioid proenkephalin pathway |
| P05917 | Opioid proopiomelanocortin pathway |
| P04391 | Oxytocin receptor mediated signaling pathway |
| P05734 | Synaptic_vesicle_trafficking |
| P04394 | Thyrotropin-releasing hormone receptor signaling pathway |
