# Supplementary figures and images for: Identification of putative baroreceptors in human aortic arch by histological and omics analyses
Source: Hypertens Res. 2025 May 7;48(7):2083–94. doi: 10.1038/s41440-025-02217-9 (PMC12229889; doi:10.1038/s41440-025-02217-9)

## Slide 1
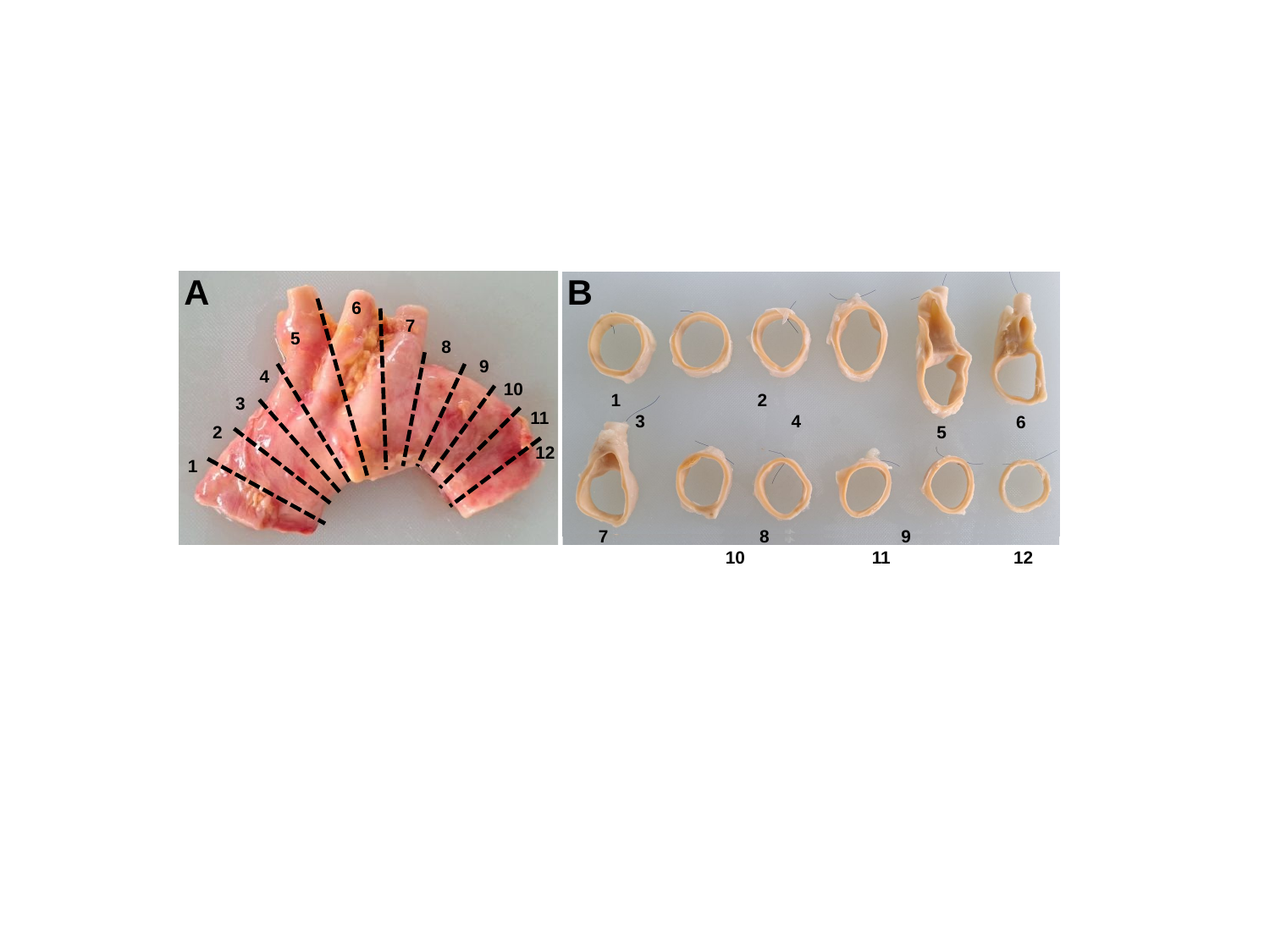

A
B
6
7
5
8
9
4
10
1 	 2 	 3	 4
3
11
6
2
5
12
1
7 	 8 	 9		10 	 11 	 12

Supplement: Supplementary file 4 — Supplementary Figure1 [file 41440_2025_2217_MOESM4_ESM.pptx]

## Slide 1
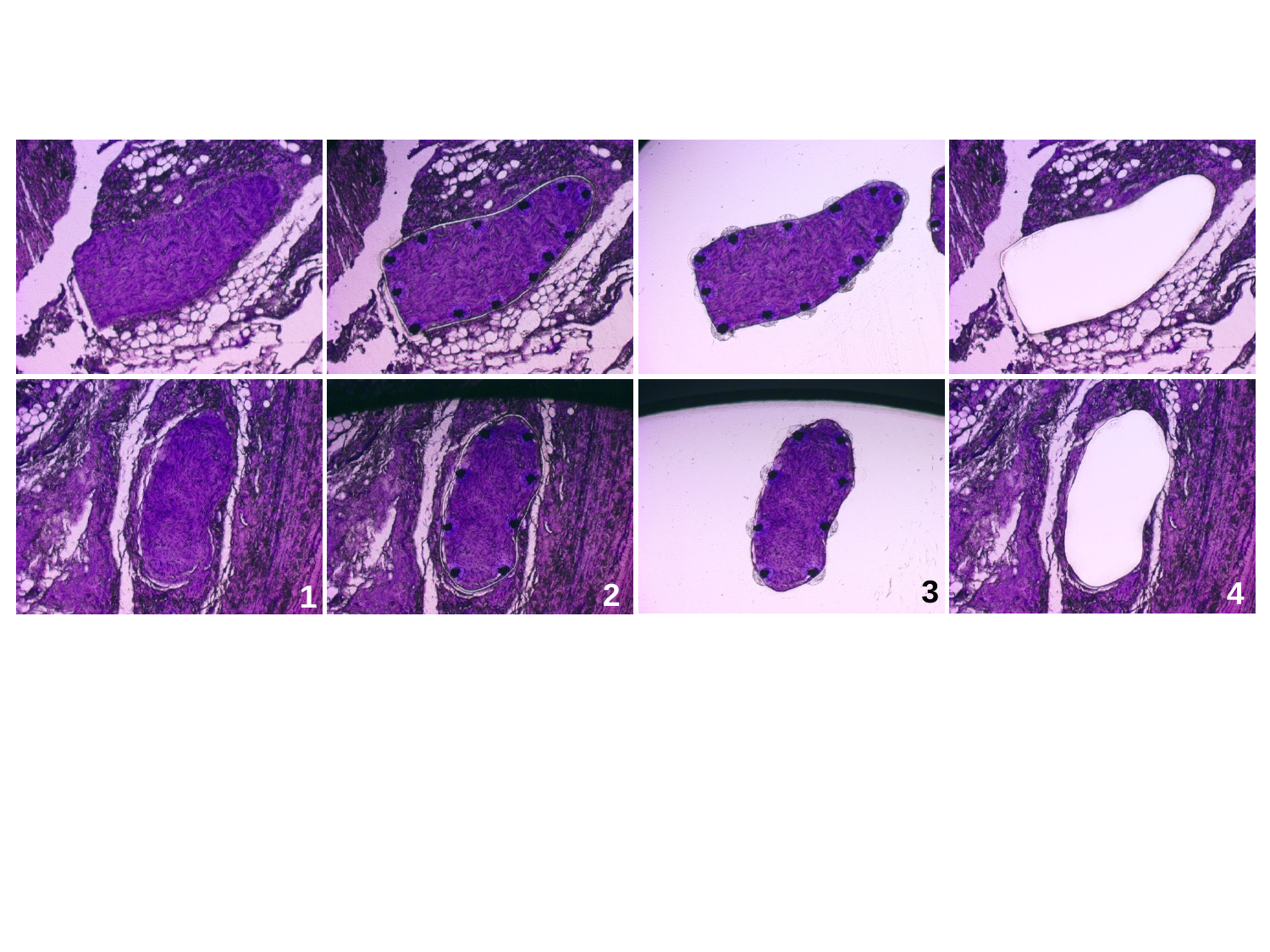

3
4
2
1

Supplement: Supplementary file 5 — Supplementary Figure2 [file 41440_2025_2217_MOESM5_ESM.pptx]
