# Supplementary material for: Identification of putative baroreceptors in human aortic arch by histological and omics analyses
Source: Hypertens Res. 2025 May 7;48(7):2083–94. doi: 10.1038/s41440-025-02217-9 (PMC12229889; doi:10.1038/s41440-025-02217-9)
Supplement: Supplementary file 6 — Supplementary Figure3 [file 41440_2025_2217_MOESM6_ESM.pptx]

## Slide 1
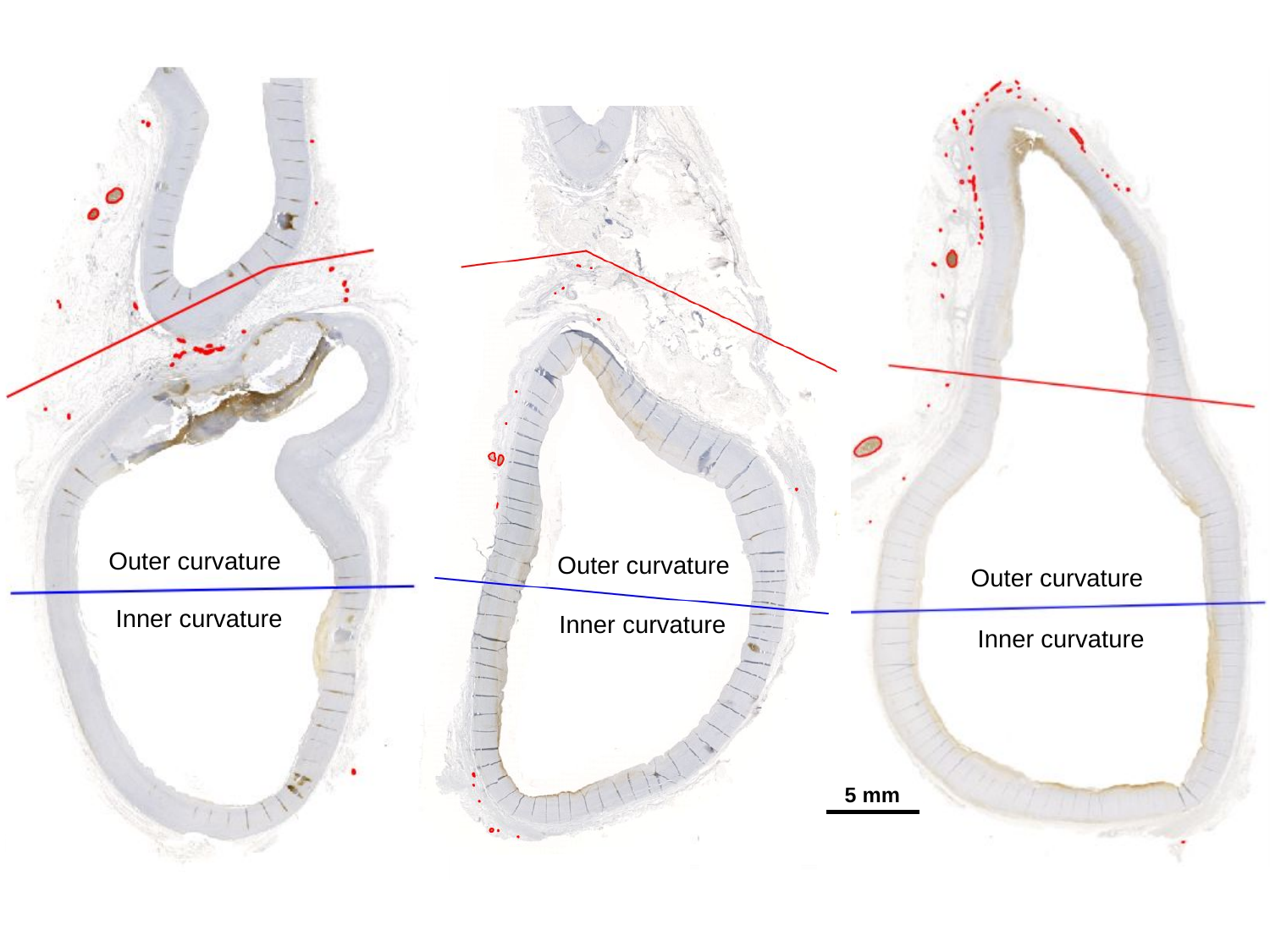

Outer curvature
Outer curvature
Outer curvature
Inner curvature
Inner curvature
Inner curvature
5 mm
