# Supplementary material for: Identification of putative baroreceptors in human aortic arch by histological and omics analyses
Source: Hypertens Res. 2025 May 7;48(7):2083–94. doi: 10.1038/s41440-025-02217-9 (PMC12229889; doi:10.1038/s41440-025-02217-9)
Supplement: Supplementary file 7 — Supplementary Figure Legend [file 41440_2025_2217_MOESM7_ESM.docx]

**Supplementary Fig. 1** Example of the human aortic arch used in the study. (A) Image of explanted aortic arch; (B) Corresponding segments used for the analysis. A suture was inserted in each segment to distinguish between the upper and lower curvatures.

**Supplementary Fig. 2** Laser Capture Microdissection. The images show the microdissection procedure for the nerve areas. Tissue samples were mounted on PEN membrane glass slides and stained with cresyl violet. The first image is of the microscope slide before LCM. The second image is of the microscope slide after the UV laser (the thin white line where the nerve is cut out) and IR laser (black spots inside the nerve on the border to the UV cut). The third image is of the cap, where the excised nerves are pasted through the IR laser. The fourth image is the remaining area after the nerve excision.

**Supplementary Fig. 3** Quantification of nerve distribution along the aortic arch by tyrosine hydroxylase (TH) staining. The blue line indicates the separation between the outer and inner curvatures. The red line indicates the separation of the aortic branch of the brachiocephalic, left common carotid and left subclavian arteries, which were excluded.
